# Feeding practices and nutrient content of complementary meals in rural central Tanzania: implications for dietary adequacy and nutritional status

**DOI:** 10.1186/s12887-015-0489-2

**Published:** 2015-11-06

**Authors:** Kissa B. M. Kulwa, Peter S. Mamiro, Martin E. Kimanya, Rajab Mziray, Patrick W. Kolsteren

**Affiliations:** Department of Food Safety and Food Quality, Ghent University, Coupure Links 653, 9000 Ghent, Belgium; Department of Food Science and Technology, Sokoine University of Agriculture, P.O. Box 3006 Chuo Kikuu, Morogoro, Tanzania; Nelson Mandela African Institute of Science and Technology, P.O. Box 447, Arusha, Tanzania; Tanzania Food and Drugs Authority, P.O. Box 77150, Dar-es-Salaam, Tanzania; Department of Public Health, Institute of Tropical Medicine, Nationalestraat 155, 2000 Antwerp, Belgium

**Keywords:** Tanzania, Complementary foods, Feeding practices, Energy, Iron, Zinc

## Abstract

**Background:**

Stunting and micronutrient deficiencies are significant health problems among infants and young children in rural Tanzania. Objective of the study was to assess feeding practices, nutrient content of complementary meals, and their implications for dietary adequacy and nutritional status.

**Methods:**

A cross-sectional study was conducted in six randomly selected villages in Mpwapwa District, Tanzania during the post-harvest season. Information on feeding practices, dietary consumption and anthropometric measurements of all infants below the age of one year were collected. Forty samples of common meals were collected and analysed for proximate composition, iron, zinc and calcium. Results were expressed per 100 g dry weight.

**Results:**

Energy, protein and fat content in porridge ranged from 40.67–63.92 kcal, 0.54–1.74 % and 0.30-2.12 %, respectively. Iron, zinc and calcium contents (mg/100 g) in porridge were 0.11–2.81, 0.10–3.23, and 25.43-125.55, respectively. Median portion sizes were small (porridge: 150–350 g; legumes and meats: 39–90 g). Very few children (6.67 %) consumed animal-source foods. Low meal frequency, low nutrient content, small portion size and limited variety reduced the contribution of meals to daily nutritional needs.

**Conclusions:**

Findings of the study highlight inadequate feeding practices, low nutritional quality of meals and high prevalence of stunting. Feasible strategies are needed to address the dietary inadequacies and chronic malnutrition of rural infants.

**Electronic supplementary material:**

The online version of this article (doi:10.1186/s12887-015-0489-2) contains supplementary material, which is available to authorized users.

## Background

Widespread undernutrition in low-income countries continues to exert enormous cost in terms of survival among infants and young children [1, 2]. Chronic undernutrition (defined as stunting) and micronutrient deficiencies are significant health problems among infants and young children in Tanzania. Prevalence of stunting among children aged 6–59 months in the 2005 and 2010 national surveys was 37.7 % and 42 %, respectively [3, 4]. Children in rural areas were more affected than their urban counterparts. Coexistence of micronutrient deficiencies with undernutrition has been demonstrated in cross-sectional studies [5, 6]. National data has also shown inadequate consumption of micronutrient-rich foods. Proportion of children (6–35 months-old) who consumed iron-rich foods was 29.8 %, whereas that of vitamin A-rich foods was 61.5 % [4]. Inadequate dietary intakes and poor feeding practices directly affect the nutritional status of children in the country. This situation is aggravated by household food insecurity.

Households in rural Tanzania depend on rain-fed, small subsistence farming for their livelihoods. Rainfall variability (e.g. timing, amount, frequency, patterns), widespread in semi-arid areas of the country, affects the timing of crop harvests and amount of food stocks in central regions (Dodoma, Singida). Dwindling food stocks, increasing food prices and seasonal shifting of maternal workload towards casual labour are apparent during the post-harvest season [7]. It was reported that 45–55 % of households in central regions were food insecure in 2006, whereas over one-third of households with tenuous access to food were reported in 2010 [7]. Proportion of households receiving food aid in Dodoma was 66.6 %.

These challenges demonstrate that many households in Dodoma are vulnerable to food insecurity. This situation provided a context in which to evaluate the extent to which household dietary vulnerability modifies feeding practices, diets and nutritional status among infants and young children. The present study was undertaken to assess feeding practices, nutrient content of complementary meals, and their implications for dietary adequacy and nutritional status in rural Dodoma.

## Methods

The study was conducted in Dodoma Region, central Tanzania Mainland. Mpwapwa District was selected by simple random sampling. The district lies between 915 and 1,200 meters above sea level. It covers an area of 7,485 square kilometres and has a total population of 253,602 [8]. The district is characterised by a long dry season (May to mid-November), a short single wet season (November to March) and monthly rainfall variability of between 50 and 300 mm. Farmers experience single staggered harvest between mid-March and June. Subsistence farming and traditional rearing of animals are the primary economic activities [9].

A cross sectional study was conducted in six randomly selected villages. All households with infants below the age of one year were recruited. A total of 496 infants participated in the study. Purpose and nature of the study activities were explained to parents and those who agreed to participate gave verbal informed consent. Ethical clearance was obtained from the National Institute of Medical Research, Tanzania.

Household, maternal and infant characteristics were collected using a structured questionnaire (See Additional file 1). Household and maternal characteristics included household size, type of household, eating frequency, status of household food sufficiency between seasons, maternal age, education and place where the study infant was delivered. Mothers provided information on infant breastfeeding and complementary feeding practices. Feeding practices were compared to WHO infant and young child feeding (IYCF) recommendations and indicators [10, 11]. Four core IYCF indicators were used, namely exclusive breastfeeding under 6 months (i.e. *Proportion of infants 0–5 months of age who are fed exclusively with breast milk*), introduction of solid, semi-solid or soft foods (i.e. *Proportion of infants 6–8 months of age who receive solid, semi-solid or soft foods*), minimum dietary diversity (i.e. *Proportion of children 6–23 months of age who receive foods from 4 or more food groups*), and minimum meal frequency (i.e. *Proportion of breastfed and non-breastfed children 6–23 months of age, who receive solid, semi-solid, or soft foods including milk feeds for non-breastfed children the minimum number of times or more*). Our assessment of IYCF indicators minimum dietary diversity and minimum meal frequency is limited to infants 6–11 months-old instead of the recommended 6–23 months-old. This limits our age-appropriate conclusion regarding these indicators.

Children were weighed with minimum clothing using an infant-hanging weighing scale (Salter model 235, CMS Weighing Equipment, London). Child’s recumbent length was taken using a portable wooden infant/child length board (Shorr Productions, Perspective Enterprises, Missouri). Standardised anthropometric procedures [12] were observed. Nutritional status indices (Z-scores for length-for-age [LAZ], weight-for-length [WLZ], weight-for-age [WAZ]) were computed using World Health Organisation (WHO) 2005 Child Growth Standards in the ANTHRO Software v3.0.1 (ANTHRO, WHO, Geneva). Indicators of nutritional status (stunting, wasting, underweight) were defined as Z-scores below −2 standard deviations (SD) of the median values of the reference data.

Chi-squared test for categorical variables and bivariate analysis for continuous variables were used to test if there were any significant associations or relations between nutritional status and feeding practices indicators.

Food intake was assessed by an interactive 24-h dietary recall [13]. Mothers recalled all foods and fluids consumed by their children during the previous 24 h, ingredients and amounts used in meal preparation and quantities of consumed foods/fluids. Calibrated cylinders, digital food weighing scales (BOECO BEB 61, 5 kg capacity 1 g precision; Hamburg, Germany) and visual aids of fresh foods were used to facilitate quantity estimation. Frequently consumed meals were identified and information on ingredients and preparation methods were compiled in a meal preparation guide. Amounts consumed (i.e. portion size in grams per meal) were recorded and expressed as median values. In order to standardise meals preparation for sample collection, focus group discussions (*n* = 6) were conducted to reach an agreement on common ingredients, amounts, preparation and cooking methods. After consensus, a meal preparation guide was developed and 80 mothers who participated in the 24-h dietary recall were randomly recruited to prepare the meals in small groups [14]. Ingredients were obtained locally. Local preparation and cooking practices were maintained. Cooking time was also documented.

After cooking, five samples per meal were weighed (BOECO BLC-3000, 3 kg capacity, 1 g precision; Hamburg, Germany) and collected for laboratory analysis. Because calcium content in water contributes to calcium content in meals and dietary intake [15], water used for cooking was randomly collected from six community sources for calcium analysis. Underground protected wells were the major sources of water for majority of the households.

Five hundred grams of each meal was packed in labelled trace element-free air-tight plastic containers (for semi-solid or solid) and bottles (for fluids). Water samples (500 ml) were packed in glass bottles and sealed. The samples were transported in ice-packed cooler boxes to the laboratory of the Department of Food Science and Technology, Sokoine University of Agriculture, Morogoro. Meal samples were kept at −18 °C and water samples at 5 °C for 3 days then transported in ice-packed cooler boxes to the Tanzania Food and Drugs Authority (TFDA) laboratory, Dar-es-Salaam. Meal samples were stored at −18 °C for 1 month awaiting analysis. Water samples were analysed for calcium within 14 days from the time of collection. On the day of analysis, meal samples were thawed at room temperature and homogenised using a stainless-steel blender (Copley Scientific, Nottingham, UK). For each nutrient parameter, 100 gram of homogenised sample was taken for analysis. All analyses were carried out in independent duplicate samples. Values were reported as mean ± standard deviation.

Proximate composition of samples was carried out using the AOAC Official Methods of Analysis [16], with analysis of moisture (method 925.04), ash (method 938.08), fat (method 954.02), crude fibre (method 985.29) and protein (method 981.10). Moisture content was determined by air drying in oven at 105 °C. Ash was determined after combustion of sample in a muffle furnace (Carbolite CWF 1200) at 550 °C for 4–6 h. Crude fat was obtained using a Soxhlet method. Crude fibre was determined after digestion with sulphuric acid and sodium hydroxide and ashing in muffle furnace at 600 °C. Crude protein was determined by Auto-Kjedahl method (Kjeltec 2300, FOSS). Conversion of nitrogen values to protein was calculated using a factor of 6.25 for meat, fish, maize and beans; 6.38 for milk; 6.31 for millet; 5.95 for rice; and 6.25 for other meals where a conversion factor is not specified [17]. Available carbohydrate was calculated by percentage difference between 100 and sum of the fat, protein, moisture, ash and fibre values. Energy content of sample meal was calculated using the Atwater factors [18] and expresses as kcal/100 g dry weight. Proximate composition results (%/100 g) were expressed per 100 g dry weight. Energy density was calculated by dividing energy content (kcal) to weight (g) of a meal.

Concentrations of calcium, iron and zinc were determined individually in the aliquots of air-dried, ashed acid-dissolved samples using Graphite-Furnace Atomic Absorption Spectrometry (GFAAS, 6300, Shimadzu, Tokyo, Japan) according to AOAC methods [16]. For calcium determinations, lanthanum chloride (1 % w/v) was added to both standards and samples to suppress interference from phosphorus [19, 20]. Mineral content less than 0.06 mg/100 g were categorised as trace according to suggested analytical limits [17]. Standards were prepared from stock standard solutions of zinc, iron and calcium (Scharlau, Scharlab SL, Spain). Results, mg/100 g, were expressed per 100 g dry weight.

To minimise risk of contamination, all glassware and plastic ware were acid-washed and rinsed with deionised water before use. Sterile disposable powder-free plastic gloves were worn when handling samples. All chemicals and solvents used were of analytical grade. Reagent blanks helped to monitor purity of the reagent used. Duplicate measurements falling within 10 % of their mean were accepted to be showing satisfactory agreement. Analysis was repeated if the agreement was outside 10 % of the mean for the duplicates [21]. Analyses of in-house reference materials were used for quality assurance. Maize flour was used for analyses of moisture, ash and protein; soybean oil was used as in-house control sample for fat; whole wheat flour for fibre; and rice flour (Standard Reference Material 1568a) for the minerals.

## Results

Of the 496 recruited infants (0–11 months-old), 99.6 % were breastfed 24 h before the survey. Sixty percent of infants below the age of 6 months (*n* = 175) had received liquids and semi-solid foods earlier than the recommended age of 6 months. Mean age of introduction of complementary foods was 3.30 ± 1.45 (range: 1–6). Majority (93 %) of the infants aged 6–8 months were receiving soft, semi-solid or solid foods. Mean number of meals consumed including snacks was 1.74 ± 0.73 (range: 1–4), with 6–8 months-old infants having lower frequency (1.66 ± 0.65) than 1.84 ± 0.71 for infants aged 9–11 months. Mean number of individual food items consumed (i.e. food variety) was 2.27 ± 1.43 (range: 1–8). Very few children (6.67 %) consumed animal-source foods. Proportion of infants (6–11 months-old) who met the WHO minimum dietary diversity criterion of 4 or more food groups was 4.6 %. The infants consumed 1 to 5 food groups. Prevalence of stunting and wasting was 33.7 % and 2.4 %, respectively. There were no significant associations or relations between nutritional status and any of the IYCF indicators. Other infant, maternal and household characteristics are shown in Table 1.

**Table 1:** Characteristics of infants 0–11 months of age (*n* = 496), mothers and households participating in the study

Variable	*n* (%)	mean (SD)	Variable	*n* (%)	mean (SD)
Infant			Consumption of solid, semi-solid, soft foods at 6–8 mo		
Age (months)					
0-5	175 (35.3)		Breast milk alone	11 (7)	
6-8	157 (31.7)		Breast milk, other foods and fluids	146 (93)	
9-11	164 (33.1)		Infant dietary diversity (*n* = 390)		1.66 (0.88)
Sex			1-3 food groups	372 (95.4)	
Male	247 (49.7)		4 or more	18 (4.6)	
Female	249 (50.3)		*Maternal*		
Stunting (*n* = 492)			Maternal age		26.57 (7.16)
All	166 (33.7)	−1.48 (1.32)^a^	Maternal education (years)		4.84 (3.05)
0-5	37 (7.5)		No education	198 (39.9)	
6-8 mo	48 (9.7)		Primary	291 (58.7)	
9-11 mo	81 (16.5)		Secondary and above	7 (1.4)	
Wasting (*n* = 492)			Place infant was delivered		
All	12 (2.4)	0.47 (1.33)^a^	Health facility	290 (58.5)	
0-5	4 (0.8)		Home	206 (41.5)	
6-8 mo	6 (1.2)		*Household*		
9-11 mo	2 (0.4)		Household size		5.26 (1.96)
Underweight (*n* = 492)			Type of household		
All	59 (12.0)	−0.61 (1.20)^a^	Male-headed	412 (83.1)	
0-5	14 (2.8)		Female-headed	84 (16.9)	
6-8 mo	20 (4.1)		Eating frequency		2.14 (0.54)
9-11 mo	25 (5.1)		1	42 (8.5)	
Feeding frequency		1.74 (0.73)	2	342 (69.0)	
1	172 (42.5)		3	112 (22.6)	
2	169 (41.7)		If food is sufficient between seasons		
3	62 (15.3)		Yes	153 (30.8)	
4	2 (0.5)		No	343 (69.2)	

^a^Mean and SD of the Z-scores for length-for-age (LAZ), weight-for-length (WLZ) and weight-for-age (WAZ), respectively

Porridge was the main complementary meal. Common types of flour were maize, sorghum, pearl millet and finger millet. See Additional file 2 for description of porridge ingredients and preparation methods. Flour was mixed with water in a flour to water ratio ranging from 1:4 to 1:9. Cow’s milk was added in porridge or consumed as a beverage after dilution with water supposedly to make it ‘light’ for infants to consume. Other meals included staple eaten together with a relish (stew or sauce). The staples included stiff porridge (or *ugali* in Kiswahili) and white rice. Relish was based on beef, fish, sardines, fermented milk, kidney beans and green-leafy vegetables. See Additional file 3 for description of staple and relish ingredients and preparation methods. Relish was prepared as a family meal, from which a portion was served to the infant. Being a dry season, fresh vegetables were obtained from locally-irrigated plots, whereas dried vegetables were obtained from households’ stock of previous harvest. The vegetables are usually harvested fresh during the rainy season, de-stalked, open sun-dried and stored in air-tight clay pots until consumption during the dry season.

Proximate composition of porridge samples and portion sizes estimated from the 24-h dietary recall among infants aged 6–11 months are shown in Table 2. Porridge samples had high moisture content. Porridge containing groundnuts or cow’s milk had slightly higher protein content than others. Fat content was slightly high in composite porridge and whole maize porridge made with groundnuts, cow’s milk or sunflower oil. Composite porridge contained the highest amount of calculated energy.

**Table 2:** Proximate composition and energy content of porridge varieties

Porridge type	Median portion^a^	*n* ^b^	Moisture [ %/100 g]	Energy [kcal/100 g]	Ash	Fat	Protein	Available Carbohydrate	Fibre
					[ % per 100 g dry weight]				
Whole maize flour, Sugar	215	58	83.94 ± 0.59	53.07	0.85 ± 0.01	0.91 ± 0.02	1.00 ± 0.01	10.21	3.09 ± 0.03
Whole maize flour, Groundnuts, Salt	215	58	83.25 ± 0.58	54.55	1.05 ± 0.01	1.20 ± 0.02	1.33 ± 0.01	9.61	3.56 ± 0.04
Whole maize Groundnuts, Salt, Sugar	215	58	83.82 ± 0.59	51.40	0.93 ± 0.01	0.95 ± 0.02	1.19 ± 0.01	9.53	3.58 ± 0.04
Whole maize flour, Baobab flour	215	58	84.16 ± 0.60	47.45	0.96 ± 0.01	0.76 ± 0.01	0.96 ± 0.01	9.19	3.97 ± 0.04
Whole maize flour, Baobab flour, Sugar	215	58	84.40 ± 0.61	47.40	0.89 ± 0.01	0.68 ± 0.01	0.59 ± 0.01	9.74	3.70 ± 0.04
Whole maize flour Sunflower oil, Salt	215	58	83.54 ± 0.58	61.70	0.98 ± 0.01	2.12 ± 0.04	0.99 ± 0.01	9.656	2.71 ± 0.03
Composite flour (Whole maize flour, Finger millet flour, Sardines, Groundnuts, Salt)	315^**c**^	10	82.24 ± 0.57	63.92	0.97 ± 0.01	1.84 ± 0.04	1.35 ± 0.01	9.88	3.72 ± 0.04
Whole maize flour, Cow’s milk, Salt	215	79	86.30 ± 0.60	45.43	0.83 ± 0.01	1.17 ± 0.02	1.04 ± 0.01	7.69	2.97 ± 0.03
Dehulled maize flour, Salt	245	74	85.74 ± 0.60	49.68	0.57 ± 0.00	0.48 ± 0.01	0.66 ± 0.01	10.69	1.86 ± 0.02
Dehulled maize flour, Groundnuts, Sugar	245	74	84.15 ± 0.59	52.37	0.83 ± 0.01	0.73 ± 0.01	1.04 ± 0.01	10.41	2.85 ± 0.03
Dehulled maize flour, Cow’s milk, Salt	245	74	88.37 ± 0.63	40.67	0.68 ± 0.00	0.81 ± 0.02	1.29 ± 0.01	7.05	1.80 ± 0.02
Dehulled and soaked maize flour, Salt	220	27	86.48 ± 0.61	47.54	0.54 ± 0.00	0.30 ± 0.01	0.54 ± 0.01	10.68	1.46 ± 0.02
Dehulled and soaked maize flour, Groundnuts, Salt	220	27	86.53 ± 0.61	47.18	0.57 ± 0.00	0.66 ± 0.01	1.02 ± 0.01	9.30	1.93 ± 0.02
Dehulled and soaked maize flour, Baobab, Sugar	220	27	86.68 ± 0.61	46.00	0.82 ± 0.01	0.51 ± 0.01	0.71 ± 0.01	9.65	1.63 ± 0.02
Dehulled and soaked maize flour, Cow’s milk, Sugar	220	27	86.85 ± 0.62	49.56	0.51 ± 0.00	0.63 ± 0.01	0.99 ± 0.01	9.98	1.03 ± 0.01
Whole sorghum flour, Salt	187.5	44	84.53 ± 0.59	47.87	0.98 ± 0.01	0.76 ± 0.01	1.47 ± 0.01	8.80	3.47 ± 0.04
Whole sorghum flour, Groundnuts, Salt	187.5	44	84.61 ± 0.60	48.03	0.86 ± 0.01	1.10 ± 0.02	1.74 ± 0.02	7.79	3.90 ± 0.04
Whole pearl millet flour, Salt	227.5	10	84.25 ± 0.59	45.46	0.97 ± 0.01	0.79 ± 0.02	1.35 ± 0.01	8.24	4.41 ± 0.05
Whole pearl millet flour, Groundnuts, Salt	227.5	10	83.95 ± 0.59	45.27	0.98 ± 0.01	0.85 ± 0.02	1.48 ± 0.01	7.93	4.81 ± 0.05
Whole finger millet flour, Sugar	227.5	10	83.47 ± 0.58	46.16	1.03 ± 0.01	0.86 ± 0.02	1.50 ± 0.01	8.12	5.03 ± 0.06
Fresh cow’s milk, Water, Sugar	150	10	86.83 ± 0.62	66.89	0.54 ± 0.00	3.27 ± 0.06	2.29 ± 0.02	7.07	NA

Data are expressed as mean ± SD on a dry-weight basis. NA-not analysed

^a^ Infant median portion sizes in grams per meal recorded from the 24-hour dietary recall among infants aged 6–11 months

^b^ Number of infants reported to have consumed the meal on the day of the 24-hour dietary recall. Infants who had 2 or more meals per day consumed same or a different type of porridge

^c^Consumed by 9–11 months-old infants only

Table 3 presents proximate composition for staples and accompanied relish. Meal portion sizes estimated from the 24-h dietary recall are also shown in Table 3. Protein content was higher in whole maize *ugali* than other staples. Relish based on beef and fish contained higher amounts of protein, fat and energy compared to others. Inclusion of groundnuts in jute mallow leaves contributed to slight increase in fat compared to a similar relish without groundnuts.

**Table 3:** Proximate composition and energy content of cooked staple and accompanied relish

Meal type	Median portion^a^	*n* ^b^	Moisture [ %/100 g]	Energy [kcal/100 g]	Ash	Fat	Protein	Available Carbohydrate	Fibre
					[ % per 100 g dry weight]				
Staple									
Whole maize *ugali*	110	66	62.51 ± 0.44	138.68	0.89 ± 0.01	1.28 ± 0.02	4.38 ± 0.04	27.41	3.53 ± 0.04
Dehulled maize *ugali*	140	66	66.15 ± 0.46	129.01	0.60 ± 0.00	0.59 ± 0.01	2.08 ± 0.02	28.85	1.74 ± 0.02
Dehulled and soaked maize *ugali*	140	66	69.12 ± 0.48	120.44	0.53 ± 0.00	0.39 ± 0.02	0.28 ± 0.00	28.96	0.73 ± 0.01
Whole sorghum stiff *ugali*	110	66	63.65 ± 0.45	129.33	0.97 ± 0.01	0.96 ± 0.02	3.85 ± 0.04	26.31	4.26 ± 0.05
Rice cooked	105	2	63.84 ± 0.45	145.03	0.88 ± 0.01	1.61 ± 0.03	4.18 ± 0.04	28.45	1.04 ± 0.01
Relish									
Beef relish	65	10	65.86 ± 0.46	190.13	2.05 ± 0.01	12.91 ± 0.25	18.05 ± 0.16	0.44	0.69 ± 0.01
Fish relish	90	2	62.57 ± 0.44	191.49	2.45 ± 0.02	10.44 ± 0.20	23.84 ± 0.22	0.54	0.16 ± 0.00
Sardines relish	82.5	4	63.59 ± 0.44	173.45	2.00 ± 0.01	7.48 ± 0.14	26.20 ± 0.24	0.32	0.40 ± 0.00
Fermented cow’s milk	150	3	89.20 ± 0.62	58.30	1.26 ± 0.01	4.03 ± 0.08	2.70 ± 0.02	2.81	NA
Bean relish with tomato	90	12	71.35 ± 0.49	98.13	2.18 ± 0.01	2.06 ± 0.04	3.70 ± 0.03	16.20	4.51 ± 0.05
Bean relish without tomato	72.5	12	71.22 ± 0.49	95.75	2.19 ± 0.01	1.47 ± 0.03	4.68 ± 0.04	15.95	4.49 ± 0.05
Chinese cabbage	54	44	71.84 ± 0.50	106.32	3.11 ± 0.02	8.21 ± 0.16	2.35 ± 0.02	5.75	8.74 ± 0.10
Sweet potato leaves	54	44	74.47 ± 0.52	73.41	4.14 ± 0.03	4.17 ± 0.08	1.27 ± 0.01	7.69	8.26 ± 0.10
Fresh Cowpea leaves	54	44	71.35 ± 0.49	129.61	3.04 ± 0.02	9.78 ± 0.19	4.75 ± 0.04	5.64	5.44 ± 0.06
Dried Cowpea leaves	50	44	75.04 ± 0.53	111.76	2.34 ± 0.01	9.81 ± 0.19	3.08 ± 0.03	2.79	6.94 ± 0.08
Pumpkin leaves	54	44	74.80 ± 0.52	85.28	5.08 ± 0.03	6.27 ± 0.12	3.19 ± 0.03	4.01	6.64 ± 0.08
Jute mallow leaves with groundnuts	54	44	76.80 ± 0.54	66.56	4.83 ± 0.03	1.98 ± 0.04	2.52 ± 0.03	9.67	4.21 ± 0.05
Jute mallow leaves without groundnuts	50	44	75.47 ± 0.53	64.34	5.62 ± 0.03	1.41 ± 0.03	3.42 ± 0.03	9.49	4.59 ± 0.05
Kale leaves	50	44	72.56 ± 0.51	103.96	4.88 ± 0.03	7.93 ± 0.15	2.17 ± 0.02	5.98	6.48 ± 0.07

Data are expressed as mean ± SD on a dry-weight basis. NA-not analysed

^a^Infant median portion sizes in grams per meal recorded from the 24-hour dietary recall among infants aged 6–11 months

^b^Number of infants reported to have consumed the meal on the day of the 24-hour dietary recall. Infants who had 2 or more meals per day consumed same staple, same relish or a different type of relish

Iron, zinc and calcium contents in porridge are shown in Table 4. Iron content was lowest in dehulled and soaked maize porridge and highest in whole finger millet porridge. Zinc content was highest in the composite porridge. Iron, zinc and calcium contents in staples and relish are presented in Table 5. Beef was a rich source of zinc, whereas dried jute mallow leaves contained highest amount of iron. Mean calcium levels of domestic water samples collected in the area was 120.97 mg/L (range: 115.50 – 129.02).

**Table 4:** Calcium, iron and zinc content of porridge and contribution to recommended intakes

Porridge ingredients	Calcium	Iron	Zinc	Calcium		Iron		Zinc	
	[mg/100 g dry weight]			mg/portion	% RNI^a^	mg/portion	% RNI^a^	mg/portion	% RNI^a^
Whole maize flour, Sugar, Water	25.43 ± 1.37	0.45 ± 0.02	0.29 ± 0.01	8.78	2.2	0.16	1.7	0.10	2.4
Whole maize flour, Groundnuts, Salt, Water	78.72 ± 4.25	0.57 ± 0.03	0.36 ± 0.01	28.35	7.1	0.21	2.2	0.13	3.1
Whole maize flour, Groundnuts, Salt, Sugar, Water	34.08 ± 1.84	0.48 ± 0.02	0.25 ± 0.01	11.86	3.0	0.17	1.8	0.09	2.2
Whole maize flour, Baobab flour, Water	55.98 ± 3.02	0.24 ± 0.01	0.17 ± 0.00	19.06	4.8	0.08	0.9	0.06	1.4
Whole maize flour, Baobab flour, Sugar, Water	95.13 ± 5.14	ND	0.10 ± 0.00	31.91	8.0	NA	NA	0.03	0.8
Whole maize flour, Sunflower oil, Salt, Water	106.96 ± 5.78	0.37 ± 0.02	0.17 ± 0.00	37.85	9.5	0.13	1.4	0.06	1.5
Composite flour (Whole maize, Whole finger millet, Groundnuts, Sardines	68.14 ± 3.68	1.49 ± 0.07	0.50 ± 0.01	38.12	9.5	0.83	9.0	0.28	6.8
Whole maize flour, Cow’s milk, Salt, Water	74.27 ± 4.01	0.35 ± 0.02	0.25 ± 0.01	21.87	5.5	0.10	1.1	0.07	1.8
Dehulled maize flour, Salt, Water	74.12 ± 4.00	0.24 ± 0.01	Trace	25.90	6.5	0.08	0.9	NA	NA
Dehulled maize flour, Groundnuts, Sugar, Water	72.07 ± 3.89	0.38 ± 0.02	0.20 ± 0.00	27.99	7.0	0.15	1.6	0.08	1.9
Dehulled maize flour, Cow’s milk, Salt, Water	85.33 ± 4.61	0.19 ± 0.01	Trace	24.31	6.1	0.05	0.6	NA	NA
Dehulled and soaked maize flour, Salt, Water	78.43 ± 4.24	0.08 ± 0.03	0.13 ± 0.00	23.33	5.8	0.02	0.3	0.04	0.9
Dehulled and soaked maize flour, Groundnuts, Salt, Water	87.82 ± 4.74	ND	0.19 ± 0.00	26.02	6.5	NA	NA	0.06	1.3
Dehulled and soaked maize flour, Baobab, Sugar, Water	103.96 ± 5.61	ND	ND	30.46	7.6	NA	NA	NA	NA
Dehulled and soaked maize flour, Cow’s milk, Sugar, Water	125.55 ± 6.78	ND	Trace	36.32	9.1	NA	NA	NA	NA
Whole sorghum flour, Salt, Water	81.40 ± 4.40	0.77 ± 0.04	Trace	23.61	5.9	0.22	2.4	NA	NA
Whole sorghum flour, Groundnuts, Salt, Water	103.57 ± 5.60	0.75 ± 0.04	Trace	29.89	7.5	0.22	2.3	NA	NA
Whole pearl millet flour, Salt, Water	57.57 ± 3.11	2.05 ± 0.10	0.23 ± 0.01	20.63	5.2	0.73	7.9	0.08	2.0
Whole pearl millet flour, Groundnuts, Salt, Water	97.97 ± 5.29	2.29 ± 0.11	0.35 ± 0.01	35.77	8.9	0.84	9.0	0.13	3.1
Whole finger millet flour, Sugar, Water	93.61 ± 5.06	2.81 ± 0.14	0.44 ± 0.01	35.20	8.8	1.06	11.4	0.16	4.0
Fresh cow’s milk, Water, Sugar	60.12 ± 3.25	ND	0.18 ± 0.00	11.88	3.0	NA	NA	0.04	0.9

^a^Proportion of the WHO/FAO (2004) requirements for iron (9.3 mg/day, medium bioavailability), zinc (4.1 mg/day, moderate bioavailability) and calcium (400 mg/day) for 6–11 month-old infants

Data are expressed as mean ± SD on a dry-weight basis

*ND* not detected. Trace-values less than 0.06 mg/100 g dry weight

*NA* not applicable because mineral levels were either not detected or values were trace (less than 0.06 mg/100 g dry weight)

**Table 5:** Calcium, iron and zinc content of staple and relish and contribution to recommended intakes

Meal type	Calcium	Iron	Zinc	Calcium		Iron		Zinc	
	[mg/100 g dry weight]			mg/portion	% RNI^c^	mg/portion	% RNI	mg/portion	% RNI
Staple									
Whole maize *ugali*	11.40 ± 0.62	2.04 ± 0.10	0.97 ± 0.02	4.70	1.2	0.84	9.0	0.40	9.8
Dehulled maize stiff *ugali*	8.70 ± 0.47	0.82 ± 0.04	0.87 ± 0.02	3.98	1.0	0.38	4.0	0.40	9.7
Dehulled and soaked maize *ugali*	8.70 ± 0.47	ND	0.62 ± 0.01	3.82	1.0	NA	NA	0.27	6.6
Whole sorghum *ugali*	10.50 ± 0.57	3.230 ± 0.16	0.60 ± 0.01	3.85	1.0	1.18	12.7	0.22	5.4
White rice cooked	19.10 ± 1.03	ND	0.33 ± 0.01	7.25	1.8	NA	NA	0.13	3.1
Relish and ingredients									
Beef, Tomatoes, Onions, Oil, Salt	191.70 ± 10.35	7.39 ± 0.36	1.61 ± 0.03	53.75	13.4	2.07	22.3	0.45	11.0
Dried fish, Tomatoes, Onions, Oil, Salt	178.00 ± 9.61	3.01 ± 0.15	0.11 ± 0.00	61.56	15.4	1.04	11.2	0.04	0.9
Dried sardines, Tomatoes, Onions, Oil, Salt	114.40 ± 6.18	2.93 ± 0.14	0.17 ± 0.00	34.36	8.6	0.88	9.5	0.05	1.2
Fermented cow’s milk	216.80 ± 11.71	0.08 ± 0.00	Trace	35.12	8.8	0.01	0.1	NA	NA
Beans, Tomatoes, Onions, Oil, Salt	80.40 ± 4.34	1.78 ± 0.09	0.20 ± 0.00	20.61	5.2	0.46	4.9	0.05	1.2
Bean, Onions, Oil, Salt relish	70.70 ± 3.82	1.75 ± 0.09	0.08 ± 0.00	14.96	3.7	0.37	4.0	0.02	0.4
Chinese cabbage, Tomatoes, Onions, Oil, Salt	125.30 ± 6.77	7.24 ± 0.35	0.14 ± 0.00	19.05	4.8	1.10	11.8	0.02	0.5
Sweet potato leaves, Tomatoes, Onions, Oil, Salt	106.80 ± 5.77	7.59 ± 0.37	0.06 ± 0.00	10.11	2.5	0.72	7.7	0.01	0.1
Fresh cowpea leaves, Tomatoes, Onions, Oil, Salt	103.90 ± 5.61	5.79 ± 0.28	Trace	16.64	4.2	0.93	10.0	NA	NA
Dried cowpea leaves, Tomatoes, Onions, Oil, Salt	164.90 ± 8.91	4.22 ± 0.21	Trace	20.58	5.1	0.53	5.7	NA	NA
Pumpkin leaves, Tomatoes, Onions, Oil, Salt	141.50 ± 7.64	7.04 ± 0.35	0.07 ± 0.00	19.26	4.8	0.96	10.3	0.01	0.2
Dried jute mallow leaves, Ground nuts, Salt	81.10 ± 4.38	15.11 ± 0.74	0.10 ± 0.00	11.12	2.8	2.07	22.3	0.01	0.3
Dried jute mallow leaves, Salt	136.20 ± 7.36	17.02 ± 0.83	Trace	10.55	2.6	1.32	14.2	NA	NA
Kale leaves, Tomatoes, Onions, Oil, Salt	97.80 ± 5.28	2.75 ± 0.13	0.07 ± 0.00	11.95	3.0	0.34	3.6	0.01	0.2

^a^ Proportion of the WHO/FAO (2004) requirements for iron (9.3 mg/day, medium bioavailability), zinc (4.1 mg/day, moderate bioavailability) and calcium (400 mg/day) for 6–11 month-old infants

Data are expressed as mean ± SD on a dry-weight basis

*ND* not detected. Trace-values less than 0.06 mg/100 g dry weight

*NA* not applicable because mineral levels were either not detected or values were trace (less than 0.06 mg/100 g dry weight)

## Discussion

This present study has highlighted inadequate feeding practices, low nutrient content of complementary meals, low dietary contribution to nutritional requirements and high prevalence of chronic undernutrition (i.e. stunting) among infants in rural Dodoma.

Although majority of infants were breastfeeding as recommended, many infants were introduced to liquids and foods earlier than the recommended age of 6 months. Early introduction of complementary foods is a common practice in Tanzania [4]; 60 % in this study as compared to national levels of 33.4 % and 63.5 % among 2–3 and 4–5 months-old infants, respectively. Meal frequencies including snacks were lower than the recommended values of 2–3 for 6–8 months-old and 3–4 times for 9–11 months-old breastfed infants [10]. Majority of infants aged 6–8 months met the WHO IYCF indicator of receiving semi-solid or soft foods. High prevalence of 92.3 % for introduction of solids, semi-solid or soft foods was also reported among young children in Tanzania [22]. Very few 6–11 months-old infants met the minimum dietary diversity criterion of 4 or more food groups. A similar finding was reported in Ethiopia where 6.3 % of the children (6–24 months-old) achieved the minimum dietary diversity [23]. The mean number of individual foods consumed (i.e. food variety) was low. Limited food accessibility, low availability and lack of nutritional knowledge, could have caused the observed inadequacies [24, 25].

Although moisture content of porridge samples were within 81-87 % reported in Benin [26] and Africa [27], the flour:water ratios (1:4–1:9) used in preparations were higher than those (1:2–1:3) reported in Malawi, Ghana, Ethiopia, and other Asia Pacific countries [28]. Because water content is an important determinant of levels of other food components [17], high water content in our porridge samples contributed to high moisture content and reduced nutrient content. Energy contents of porridge samples reported here were lower than those (91.0 - 130.3 kcal) indexed in the Tanzania Food Composition Tables [29] probably because of higher water content and relatively smaller amounts of sugar used in our samples than 13 - 50 g reported in Tanzania Tables. Prolonged consumption of watery or thin porridges exposes infants to inadequate energy and nutrient intakes and chronic malnutrition.

Inclusion of groundnuts, cow’s milk or baobab fruit pulp in porridge was desirable in that they enhanced energy, protein and calcium contents and overall dietary quality. The ingredients are readily available in the study area. Nevertheless, amount of ingredients used were small and milk was diluted with water before use. These factors would limit their nutritional benefits. Consumption of traditionally fermented sour milk may be nutritionally beneficial; however the milk poses a great health risk because it was not boiled. Raw milk can easily be contaminated by pathogenic bacteria if kept for too long at ambient temperature. Because the fermentation process is spontaneous and uncontrolled, quality of sour milk may be variable; affecting taste and consequently reducing amount to be consumed.

There was limited inclusion of other nutrient-dense foods (e.g. legumes, beef, fish, sardines, vegetables) in the meals. In addition, few infants consumed these foods. Low consumption of animal source foods (ASFs) has also been reported in developing countries, resulting in inadequate dietary intake and poor growth [30–32]. Low consumption may be attributed to household food insecurity, high cost of foods, or inadequate nutritional knowledge. Due to inadequate maternal knowledge, mothers withhold the foods until infants grow sufficient number of teeth for chewing. Opportunities to increase their consumption need to be promoted. These include pounding or milling, manual grinding and mashing of raw/fresh, raw/dried and cooked foods. With the exception of a composite porridge made from a mixture of cereal, legume and animal source flour, use of composite flour was rare in this area. Formulations of mixed flours have been reported to achieve a desired nutrient content and protein complementarity [33], protein digestibility and lower viscosity as compared to single cereals [34].

Cooked staples constituted a major part of a meal and were good sources of energy. Maize is the main staple in most Tanzania communities. Although sweet potatoes, cassava and round potatoes are also consumed, they were not available at the time of the study. Types of relish reported here reflect common Tanzanian diets. Kidney beans were the only legumes available during the study. Availability of sun-dried leafy vegetables ensured their supply and consumption during the dry season. Open sun-drying method, commonly practiced in the study area and central Tanzania [35], will need to be improved in order to enhance adequate nutrient retention.

Environmental factors, grain pre-treatment prior to dehulling, extraction rates of dehulling machines, leaching of minerals in water during soaking and eventual discarding of soaking water could have accounted for reduced or undetectable levels of protein, fat, fibre, iron and zinc in meals made from dehulled or dehulled and soaked cereal flours. Calcium levels in cooking water were generally high and water samples had elevated taste of hardness. Notwithstanding the addition of calcium-rich foods in porridge (e.g. cow’s milk, baobab fruit pulp), amounts of calcium in cooking water rather than calcium intrinsic to food ingredients could be responsible for the high levels in the porridge samples. It is therefore difficult to ascertain whether the enhanced calcium contents in porridge were due to water or calcium-rich foods.

Median portion sizes for porridge were slightly higher than 115 g reported for maize-based porridge and 90 g for maize *ugali* among 6–12 months infants in South Africa [36]. Overall meal portion sizes were lower than the documented gastric capacity of 249 and 285 g/meal for 6–8 and 9–11 months infants, respectively [37]. Inadequate portion sizes will most likely translate to inadequate dietary intake. Energy density (kcal/g) and portion size (g) of foods have been identified as two properties of foods that can modulate energy intake [38]. When portion sizes were expressed in amounts of energy that could be obtained per median portion, relish made from ASFs provided higher amounts than other meals. Porridge made from composite flour provided highest energy per portion. Calculated energy densities of porridges (0.41-0.64 kcal/g) were lower than the minimum densities (0.71 kcal/g, 6–8 months-old; 0.84 kcal/g, 9–11 months-old) required to meet recommended energy from complementary foods for infants receiving two meals per day [10]. Energy densities of porridge in most developing countries have been reported to be low (0.25-0.50 kcal/g) due to addition of large quantities of water to achieve a drinkable consistency [39].

When compared to energy required from complementary foods [10], relish made from ASFs will contribute more energy (26-33 %) than staple (24-30 %), porridge (6-18 %) and beans and leafy vegetables (3-13 %) to 200 kcal/day required by 6–8 months-old infants. Likewise, ASFs will contribute more energy to 300 kcal/day required by 9–11 months-old infants. Although nutritional deficits by porridge may be addressed by consumption of staple *ugali* with relish, relish portion size would need to be increased to provide sufficient energy and other nutrients. It is also imperative that caregivers increase feeding frequency and include nutrient-rich foods in porridge.

Porridge made from composite flour provided highest amounts of zinc per portion, probably because its portion size was larger and had slightly high zinc content. Relish based on jute mallow leaves or beef provided highest amounts of iron. Compared to iron and zinc requirements (9.3 mg/day and 4.1 mg/day respectively) for 6–11 months-old infants [37, 40], none of the studied infants would be able to meet more than 25 % RNI from porridge if it was consumed twice per day. A feasible option to increase iron and zinc content in porridge would be to add locally available iron- and zinc-dense foods (e.g. dried and ground jute mallow leaves, sweet potato leaves, beans, cowpeas), increase frequency of consuming these foods and increase portion sizes as the child grows.

Inadequate feeding practices and limited dietary supply observed here could have contributed to the chronic nature of malnutrition. Although the prevalence of stunting was high, there was lack of significant associations or relations between stunting and feeding practices. Limited food availability and accessibility during the post-harvest season could have aggravated this situation. The influence of seasons on decreased household food supply and limited dietary intake has also been documented [41–43].

## Conclusions

The study shows that inadequate feeding practices, low nutrient content of complementary meals, decreased dietary contribution to nutritional requirements and high prevalence of chronic undernutrition (i.e. stunting) are very common among infants in rural Dodoma during the post-harvest season. Inclusion of groundnuts, cow’s milk or oil in porridge improves energy, protein and fat contents. Composite porridge and relish based on ASFs provide higher energy, protein and fat per portion than other meals. Relish made from beef, fish, sardines, dried jute mallow leaves, sweet potato leaves, beans and cowpeas are better sources of iron, zinc and calcium than other meals. These data provide a foundation for promoting best dietary practices (increased meal frequency, inclusion of nutrient-dense foods, adequate portion sizes, increased food variety) using feasible strategies such as nutrition education and counselling.

## Additional files

Additional file 1:
**Questionnaire: Post-harvest.** (DOC 83 kb) Additional file 2:
**Description of ingredients and methods of preparing different types of porridge.** (DOC 49 kb) Additional file 3:
**Description and methods for preparation of staple and accompanied relish.** (DOC 45 kb)
