# Supplementary material for: Feeding practices and nutrient content of complementary meals in rural central Tanzania: implications for dietary adequacy and nutritional status
Source: BMC Pediatr. 2015 Nov 6;15:171. doi: 10.1186/s12887-015-0489-2 (PMC4636743; doi:10.1186/s12887-015-0489-2)
Supplement: Additional file 1: — Questionnaire: Post-harvest. (DOC 83 kb) [file 12887_2015_489_MOESM1_ESM.doc]

**Additional file 1. Questionnaire: Post-harvest**

***Interviewer: Introduce yourself and where you come from. Explain research objectives. Thank the interviewee for his/her time.***

Name of interviewer _____________________ Date of interview (dd/mm/yyyy) ___/___/___

Household ID Number _______

**A. Background information**

1. Ward 1.Mazae 2.Lumuma
2. Village 1.Kisokwe 2.Gulwe 3.Chiseyu 4.Pwaga 5.Kitati 6.Munguwi
3. First name of mother (respondent) __________________ Second name _______________
4. Age of mother _____(yrs)
5. First name of father (household head ________________ Second name _______________
6. Age of father _____(yrs)
7. Household is headed by 1. male 2. female
8. Household composition

|  | Below 2 yrs | 2 - 4.9 yrs | 5 - 14 yrs | 15 - 19 yrs | 20 - 64 yrs | ≥ 65 yrs | **Total** |
| --- | --- | --- | --- | --- | --- | --- | --- |
| **Number** |  |  |  |  |  |  |  |

1. What type of fuel does your household mainly use for cooking? 1.wood 2.charcoal 3.kerosene 4.cow-dung 5.solar 6.others: specify _____________

**Household dietary patterns**

1. During this period, how many times per day do you usually cook family meals? _____
2. Please describe the household’s habitual eating pattern during this period

| **Eating frequency per day^1^** | **Meal occasion^2^** | **Main dish** | **Regular relish^3^** |
| --- | --- | --- | --- |
|  |  |  |  |
|  |  |  |
|  |  |  |
|  |  |  |

***^1^ Frequency: 1=once 2=twice 3=three times 4=four times. ^2^ Meal occasion: 1=breakfast 2=snack 3=lunch 4=supper. ^3^ Relish: 1= legume-based 2= fish-based 3= sardines-based 4=meat-based 5=vegetable-based 6=groundnut-based 7=yoghurt-based 8=others: write on the table***

1. Are the food crops that you cultivate usually sufficient to meet family consumption needs from one harvest season to the next? 1.Yes 2.No

**Mother characteristics**

1. How many children do you have now? _____
2. Marital status 1.single 2.married/monogamous 3.married/polygamous 4.widowed 5.separated/divorced
3. For how many years have you attended school? _____
4. Which level of education did you attain? 1.None 2.Primary-incomplete 3. Primary-complete 4.Adult education 5.Secondary-completed 6.Advanced secondary-completed 7.Certificate/Diploma for any skill 8.University-level
5. Where did you deliver this child? 1. District government hospital 2. Government health centre or dispensary 3.Missionary health centre or dispensary 4.Private health centre or dispensary 5.Home 6.Others: specify ______________

**Infant characteristics**

***If children were born twins, record information about the older twin then continue with the younger twin in a different questionnaire. Request the mother to show you her child’s clinic card. Use it to verify questions 19, 20, 23, 25, 26.***

1. First name of child __________________________ Second name ____________________
2. Date of birth (dd/mm/yy) ___/____/_____
3. Verification of date of birth 1.clinic card 2.mother recall 3.others: specify ______________
4. Age (months) ______
5. Sex 1.male 2.female
6. Birth weight _____ kg
7. Birth order 1. First born 2.Second born 3. Third born 4.Fourth born 5.Fifth born 6.Others: specify ___
8. Did you take your child to growth monitoring during the last scheduled visit? 1.Yes 2.No 8=child has just been born
9. Has the child been immunized-for-age ***(check the indicated vaccination dates)***  1.Yes 2.No 9.clinic card not present

**Infant breastfeeding and complementary feeding practices**

1. How long after birth did you put the child to the breast? 1.within the first hr 2. one to 24 hrs 3.after 24 hrs
2. Are you still breastfeeding this child? 1.Yes 2.No
3. How do you breastfeed your infant? 1.on demand by the child 2.according to schedule 3.according to mother’s inclination
4. Do you sleep with your child during the night? 1.Yes 2.No
5. What fluids and foods did your child consume yesterday during the day and at night? (ORS, vitamin and mineral syrups or medicines may be given) ***(indicate one choice only)***
6. Breast milk alone
7. Breast milk, plain water
8. Breast milk, water-based drinks (herbals, ritual fluid, teas), or fruit juices
9. Breast milk, non-human milk (e.g. animal milk, infant formula)
10. Breast milk, non-human milk (e.g. animal milk, infant formula), soft, semi-solid, or solid foods
11. Breast milk, soft, semi-solid, or solid foods
12. Soft, semi-solid, or solid foods alone
13. Since birth, have you ever given your child fluid or food other than breast milk? 1.Yes 2.No

***Note: If mother answers ‘Yes’ to question 32, continue with questions 33-39. If mother answers ‘No’ in question 32, do not ask questions 34-39, and that will be the end of the interview.***

1. What fluid or food other than breast milk did you give your infant as the first food? Specify the ingredients used in preparation. At what age did you give your baby the first fluid or food? Why did you give your child such fluid or food? How do you feed these fluids & foods?

| **Fluid or food** | **Ingredients** | **Age (mo)** | **Reason for giving fluid or food** | **How fluid/food is fed** |
| --- | --- | --- | --- | --- |
|  |  |  |  |  |

1. How many times yesterday did you feed your child fluids and foods other than breast milk? ___

**Anthropometry**

1. Infant anthropometric and biochemical measurements

| Weight (kg) | | | Length (cm) | | |
| --- | --- | --- | --- | --- | --- |
| W1 | W2 | Average | L1 | L2 | Average |
|  |  |  |  |  |  |

**Infant food intake: 24-hour recall**

1. Was the child ill yesterday? 1.Yes 2.No
2. Is the child taking any nutritional supplements? 1.Yes 2.No
3. Was yesterday a usual day or was it a festivity/celebration day where you ate special foods? 1.usual 2.festivity

***If the child was given fluids, foods or snacks yesterday, continue with question 39. If the child received breast milk only, do not ask question 39.***

1. Please tell me what your child ate and drank yesterday from the time he/she woke up to the time when he/she went to sleep. Probe for:
   1. Time (e.g. 0800, etc) and meal occasion (e.g. breakfast, snack, etc)
   2. Type of meal (e.g. bean stew, porridge, etc) or fluid (e.g. orange juice, soda, etc)
   3. Ingredients used to prepare the meal or added to already-prepared meal (e.g. refined maize flour, sugar, sour milk, etc)
   4. Amount (in household measure) of ingredients used to prepare the meal/fluid or added to meal/fluid
   5. Amount (in household measure) of total meal/fluid prepared, served to child, consumed and left uneaten

| Time | Meal occasion | Type of Meal | Ingred. used | Amount of ingred. | Stand. measure | Total vol. of meal | Stand. measure | Amount served | Amount left-over | Amount consumed |
| --- | --- | --- | --- | --- | --- | --- | --- | --- | --- | --- |
|  |  |  |  |  |  |  |  |  |  |  |
|  |  |  |  |  |  |  |  |  |  |  |
|  |  |  |  |  |  |  |  |  |  |  |
|  |  |  |  |  |  |  |  |  |  |  |
|  |  |  |  |  |  |  |  |  |  |  |
|  |  |  |  |  |  |  |  |  |  |  |
|  |  |  |  |  |  |  |  |  |  |  |
|  |  |  |  |  |  |  |  |  |  |  |
|  |  |  |  |  |  |  |  |  |  |  |
|  |  |  |  |  |  |  |  |  |  |  |
|  |  |  |  |  |  |  |  |  |  |  |
|  |  |  |  |  |  |  |  |  |  |  |
|  |  |  |  |  |  |  |  |  |  |  |
|  |  |  |  |  |  |  |  |  |  |  |
|  |  |  |  |  |  |  |  |  |  |  |
|  |  |  |  |  |  |  |  |  |  |  |
|  |  |  |  |  |  |  |  |  |  |  |
