# Supplementary material for: Feeding practices and nutrient content of complementary meals in rural central Tanzania: implications for dietary adequacy and nutritional status
Source: BMC Pediatr. 2015 Nov 6;15:171. doi: 10.1186/s12887-015-0489-2 (PMC4636743; doi:10.1186/s12887-015-0489-2)
Supplement: Additional file 2: — Description of ingredients and methods of preparing different types of porridge. (DOC 49 kb) [file 12887_2015_489_MOESM2_ESM.doc]

**Additional file 2.** Description of ingredients and methods of preparing different types of porridge

| Porridge ingredients and mixing ratio [in grams or ml] | Preparation**^a^** and cooking methods |
| --- | --- |
| Whole maize, white (*Zea mays*) |  |
| Whole maize flour, Sugar, Water  (44:5:265) | Flour added to cold water, mixed thoroughly and brought to boil while stirring constantly. Porridge left to simmer (5 mins.) with occasional stirring then sugar added to taste. |
| Whole maize flour, Groundnuts, Salt, Water  (32:11.5:0.5:195) | Flour and ground/pounded groundnuts added to cold water, mixed thoroughly. Mixture brought to boil while stirring constantly, left to simmer (5 mins.) then salt added to taste. |
| Whole maize flour, Groundnuts, Salt, Sugar, Water (40:6:1:10:360) | Flour and ground/pounded groundnuts added to cold water, mixed thoroughly. Mixture brought to boil while stirring constantly and left to simmer (5 mins.), salt and sugar added to taste. |
| Whole maize flour, Baobab fruit pulp, Water (63:26:400) | Flour added to cold water, mixed thoroughly and brought to boil while stirring constantly. Porridge left to simmer (5 mins.), baobab fruit pulp added to taste while stirring, then removed from heat. |
| Whole maize flour, Baobab fruit pulp, Sugar, Water (54:23:10:300) | Flour added to cold water, mixed thoroughly and brought to boil while stirring constantly. Porridge left to simmer (5 mins.), baobab fruit pulp added to taste while stirring, then sugar added to taste. |
| Whole maize flour, Sunflower cooking oil, Salt, Water (18:5:0.25:170) | Flour added to cold water, mixed thoroughly and brought to boil while stirring constantly. Sunflower oil added and mixture left to simmer (5 mins.) then salt added to taste. |
| Composite flour (Whole maize flour, Finger millet flour, Sardines, Groundnuts), Water | Cleaned maize grains (500g), finger millet grains (400g), groundnuts (300g) and dried de-headed sardines (300g) mixed and locally milled. 75g of composite flour added to 255ml cold water, mixed thoroughly, brought to boil while stirring constantly, left to simmer for 7 mins. |
| Whole maize flour, Cow’s milk, Salt, Water  (45:100:1.5:150) | Flour added to cold water, mixed thoroughly and brought to boil while stirring constantly. Cow’s milk added, mixture left to simmer (5 mins.), salt added to taste |
| Dehulled maize, white (*Zea mays*) |  |
| Dehulled maize flour, Salt, Water  (47:0.4:300) | Flour added to cold water, mixed thoroughly, brought to boil while stirring constantly. Porridge left to simmer (3 mins.), salt added to taste. |
| Dehulled maize flour, Groundnuts, Sugar, Water (32:11:10:200) | Flour and ground/pounded groundnuts added to cold water, mixed thoroughly. Mixture brought to boil while stirring constantly and left to simmer (3 mins.) then sugar added to taste. |
| Dehulled maize flour, Cow’s milk, Salt, Water (65:155:0.5:100) | Flour added to cold water, mixed thoroughly and brought to boil while stirring constantly. Cow’s milk was added, mixture left to simmer (3 mins.) then salt added to taste. |
| Dehulled and soaked maize, white (*Zea mays*) |  |
| Dehulled and soaked maize flour, Salt, Water (24:1.5:200) | Flour added to cold water, mixed thoroughly and brought to boil while stirring constantly. Porridge left to simmer (2 mins.), salt added to taste. |
| Dehulled and soaked maize flour, Groundnuts, Salt, Water  (32:15:0.5:270) | Flour and ground/pounded groundnuts added to cold water, mixed thoroughly. Mixture brought to boil while stirring constantly, left to simmer (2 mins.), then salt added to taste. |
| Dehulled and soaked maize flour, Baobab fruit pulp, Sugar, Water (55:16:9:340) | Flour added to cold water, mixed thoroughly and brought to boil while stirring constantly. Porridge left to simmer (2 mins.), baobab fruit pulp added to taste while stirring, then sugar added to taste. |
| Dehulled and soaked maize flour, Cow’s milk, Sugar, Water (50:40:10:230) | Flour added to cold water, mixed thoroughly and brought to boil while stirring constantly. Cow’s milk added, mixture left to simmer (2 mins.) then sugar added to taste. |
| Sorghum, white (*Sorghum bicolour* (L.) Moench) |  |
| Whole sorghum flour, Salt, Water  (105:1.25:550) | Flour added to cold water, mixed thoroughly and brought to boil while stirring constantly. Porridge left to simmer (4 mins.) then salt added to taste. |
| Whole sorghum flour, Groundnuts, Salt, Water (80:50:1.25:375) | Flour and ground/pounded groundnuts added to cold water, mixed thoroughly, brought to boil while stirring constantly, simmers (4 mins.), then salt added to taste. |
| Pearl millet (*Pennisetum glaucum*) |  |
| Whole pearl millet flour, Salt, Water  (85:2:570) | Flour added to cold water, mixed thoroughly and brought to boil while stirring constantly. Porridge left to simmer (4 mins.), salt added to taste. |
| Whole pearl millet flour, Groundnuts, Salt, Water (41:29:0.5:200) | Flour and ground/pounded groundnuts added to cold water, mixed thoroughly. Mixture brought to boil while stirring constantly, left to simmer (4 mins.) then salt added to taste. |
| Finger millet, red (*Eleusine coracana*) |  |
| Whole finger millet flour, Sugar, Water  (180:15:870) | Flour added to cold water, mixed thoroughly, brought to boil while stirring constantly. Porridge left to simmer (4 mins.) with occasional stirring then sugar added to taste. |
| Fresh cow’s milk, Water, Sugar | Milk is boiled, sieved, cream removed, water added (milk:water ratio of 1:0.5) then sugar added to taste. |

**^a^** Flour preparation: whole (winnow, sort, mill); dehulled (winnow, sort, dehull, mill); dehulled and soaked (winnow, sort, dehull, wash, soak overnight 12-14 hours in cold water [grain to water 1:4], wash, drain, sun-dry, mill)
