# Supplementary material for: Feeding practices and nutrient content of complementary meals in rural central Tanzania: implications for dietary adequacy and nutritional status
Source: BMC Pediatr. 2015 Nov 6;15:171. doi: 10.1186/s12887-015-0489-2 (PMC4636743; doi:10.1186/s12887-015-0489-2)
Supplement: Additional file 3: — Description and methods for preparation of staple and accompanied relish. (DOC 45 kb) [file 12887_2015_489_MOESM3_ESM.doc]

**Additional file 3.** Description and methods for preparation of staple and accompanied relish

| Staple and relish ingredients | Preparation and cooking methods |
| --- | --- |
| **Staple** |  |
| Whole maize stiff**^a^** porridge | Flour mixed thoroughly with cold water (flour:water range from 1:3 to 1:5). Mixture brought to boil, simmers with frequent stirring to make thick porridge. Flour added gradually with constant turning to form thick paste of desired softness then addition of flour stopped. Constant turning of paste and occasional addition of little boiled hot water continued for 4-5 minutes (2-3 minutes for dehulled or soaked flour) to ensure thorough mixing, gelatinisation and cooking. Paste covered and left to cook for 3-4 minutes (2-3 minutes for dehulled or soaked flour). |
| Dehulled maize stiff porridge | Same as above |
| Dehulled and soaked maize stiff porridge | Same as above |
| Whole sorghum stiff porridge | Same as above |
| White rice cooked | Sunflower oil and salt added into boiling water, stirred, rice added (rice:water of 1:3) and left to cook until soft and water has dried or evaporated. |
| **Relish** |  |
| Beef, Tomatoes, Onions, Sunflower oil, Salt | 500g beef (lean+fat) de-boned, chopped in small pieces, washed and boiled with regular addition of water until soft. 110g chopped onions stir-fried in 50g oil, 220g sliced tomatoes added, stirred until soft, water added to make sauce, salt added to taste. Boiled beef added in mixture and left to cook (5-10 mins). |
| Fish (dried), Tomatoes, Onions, Sunflower oil, Salt | 150g dried fish soaked in warm water for 20 mins, washed drained. 52g chopped onions stir-fried in 55g oil, 300g sliced tomatoes added, stirred until soft, water added to make sauce, salt added to taste. Pieces of fish added in mixture and left to cook (12-15 mins) until soft. |
| Sardines (dried), Tomatoes, Onions, Sunflower oil, Salt | 150g sardines sorted, de-headed, soaked in hot water for 15 mins, washed and drained. 52g chopped onions stir-fried in 40g oil, 240g sliced tomatoes added and stirred until soft. Sardines added, mixed, water added to make soup, salt added to taste and left to cook (10-15 mins) until soft. |
| Fermented cow’s milk**^b^** | Unboiled cow’s milk sieved, kept in a local gourd, covered, kept in a warm kitchen corner and left to ferment for 2-3 days. When ready, it was stirred with a wooden stick to a desired consistency. |
| Beans (*Phaseolus vulgaris* L.), Tomatoes, Onions, Sunflower oil, Salt | 500g winnowed, sorted, washed, drained and boiled with regular addition of water until soft. 32g chopped onions stir-fried in 15g oil, 90g sliced tomatoes added and stirred until soft. Boiled beans added, mixed, water added to make soup, salt added to taste, and left to cook (7-10 mins). |
| Beans (*Phaseolus vulgaris* L.), Onions, Sunflower oil, Salt | 500g winnowed, sorted, washed, drained, and boiled with regular addition of water until soft. 77g chopped onions stir-fried in 5g oil until soft, boiled beans added, mixed, water added to make soup, salt added to taste and left to cook (7-10 mins). |
| Chinese cabbage (*Brassica rapa* L. var. chinensis), Tomatoes, Onions, Sunflower oil, Salt | Fresh leaves de-ribbed, washed and finely chopped. 80g chopped onions were stir-fried in 68g oil, 240g sliced tomatoes added and stirred until soft (little water added occasionally). 450g chopped leaves were added, mixed, salt added to taste, and left to cook (10-15 mins.) until soft. |
| Sweet potato leaves (*Ipomea batatas* L.), Tomatoes, Onions, Sunflower oil, Salt | Fresh leaves had the small upper section destalked and thin outer skin peeled off. Leaves were washed, drained and cut. 85g chopped onions, 240g sliced tomatoes, 7g oil, and 400g cut leaves were put in a pot. Salt added to taste, pot covered and put on fire. Mixture was left to boil (5-7 mins.) with occasional stirring and little addition of water if needed until soft. |
| Fresh cowpea leaves (*Vigna unguiculata* L.), Tomatoes, Onions, Sunflower oil, Salt | Fresh leaves destalked, washed, cut. 240g cut leaves added into boiling water, left to boil (30-45 mins.) while covered until soft. 105g chopped onions stir-fried in 35g oil, 186g sliced tomatoes added and stirred until soft (little water added occasionally). Boiled leaves were added, mixed, salt added to taste, and left to cook (5-10 mins). |
| Dried cowpea leaves (*Vigna unguiculata* L.), Tomatoes, Onions, Sunflower oil, Salt | 120g dried leaves soaked in water (15 mins.), washed, drained. 85g chopped onions and 135g sliced tomatoes were stir-fried in 21g oil until soft. Soaked leaves were added, salt added to taste, and left to cook (15-20 mins.) with occasional stirring to avoid sticking |
| Pumpkin leaves (*Curcubita maxima* Duchesne), Tomatoes, Onions, Sunflower oil, Salt | Fresh leaves had the outer skin peeled, a small upper section destalked, washed, chopped finely (620g), and boiled (15-20 mins.) while covered until soft. 83g chopped onions were stir-fried in 90g oil, 152g sliced tomatoes added and stirred until soft (little water added if needed). Boiled leaves were added, mixed, salt added to taste, and left to cook (5-10 mins). |
| Dried jute mallow leaves (*Corchorus olitorius* L.), Ground nuts, Salt | Dried leaves pounded, sieved to remove inedible stalks. Groundnuts pounded to obtain soft flour. 340g pounded leaves and 35g groundnut flour mixed with cold water to make a smooth liquid mixture. Mixture brought to boil with stirring to make a paste, salt added to taste, and left to boil (7-10 mins) stirring occasionally to avoid sticking. |
| Dried jute mallow leaves (*Corchorus olitorius* L.), Salt | Dried leaves pounded, sieved to remove inedible stalks. 320g pounded leaves mixed with cold water to make a smooth liquid mixture. Mixture brought to boil with occasional stirring to make a paste, salt added to taste, and left to boil (7-10 mins) with occasional stirring to avoid sticking. |
| Kale leaves (*Brassica oleracea* L. var. acephala), Tomatoes, Onions, Sunflower oil, Salt | Fresh leaves de-ribbed, washed, finely chopped. 29g chopped onions were stir-fried in 95g oil, 200g sliced tomatoes added and stirred until soft (little water added occasionally). 540g chopped leaves were added, mixed, salt added to taste, and left to cook (10-15 mins.) while covered until soft. |

**^a^**Thick porridge paste locally called *ugali*

**^b^**Spontaneously fermented. Consumed alone or with ugali or rice
